# Polyelectrolyte layer-by-layer deposition on nanoporous supports for ion selective membranes†

**DOI:** 10.1039/c8ra05580g

**Published:** 2018-09-25

**Authors:** Stephen J. Percival, Leo J. Small, Erik D. Spoerke, Susan B. Rempe

**Affiliations:** Sandia National Laboratories PO Box 5800, MS 1411 Albuquerque NM USA 87185 sperciv@sandia.gov ljsmall@sandia.gov

## Abstract

This work demonstrates that the ionic selectivity and ionic conductivity of nanoporous membranes can be controlled independently *via* layer-by-layer (LbL) deposition of polyelectrolytes and subsequent selective cross-linking of these polymer layers. LbL deposition offers a scalable, inexpensive method to tune the ion transport properties of nanoporous membranes by sequentially dip coating layers of cationic polyethyleneimine and anionic poly(acrylic acid) onto polycarbonate membranes. The cationic and anionic polymers are self-assembled through electrostatic and hydrogen bonding interactions and are chemically crosslinked to both change the charge distribution and improve the intermolecular integrity of the deposited films. Both the thickness of the deposited coating and the use of chemical cross-linking agents influence charge transport properties significantly. Increased polyelectrolyte thickness increases the selectivity for cationic transport through the membranes while adding polyelectrolyte films decreases the ionic conductivity compared to an uncoated membrane. Once the nanopores are filled, no additional decrease in conductivity is observed with increasing film thickness and, upon cross-linking, a portion of the lost conductivity is recovered. The cross-linking agent also influences the ionic selectivity of the resulting polyelectrolyte membranes. Increased selectivity for cationic transport occurs when using glutaraldehyde as the cross-linking agent, as expected due to the selective cross-linking of primary amines that decreases the net positive charge. Together, these results inform deposition of chemically robust, highly conductive, ion-selective membranes onto inexpensive porous supports for applications ranging from energy storage to water purification.

## Abstract 2

This work demonstrates that the ionic selectivity and ionic conductivity of nanoporous membranes can be controlled independently *via* layer-by-layer (LbL) deposition of polyelectrolytes and subsequent selective cross-linking of these polymer layers.

## Graphical abstract

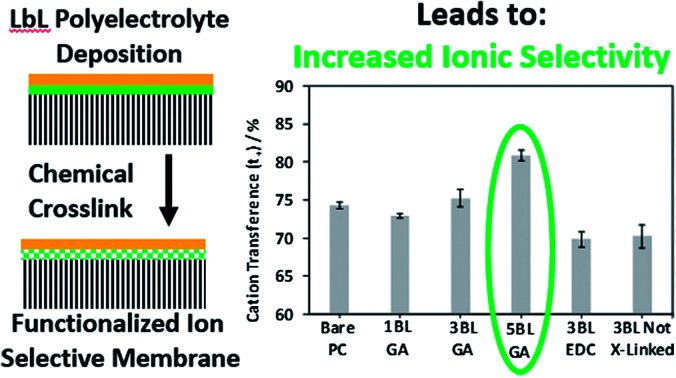

## Introduction

Nanoporous membranes offer a convenient platform for controlling ion transport.^1^ The relative ratio of pore surface area to electrolyte volume provides an opportunity to tune ion transport through the pore by controlling surface charge on the pore wall.^2,3^ Many groups have successfully leveraged a variety of responsive chemistries to alter the surface charge, and resulting ion transport, through a nanoporous membrane.^4–9^ Moreover, different ion transport behavior can be achieved through control of the nanopore shape. For example, cones can give rise to different degrees of ion rectifying behaviors normally absent in simple cylindrical nanopores, an important factor for overall control of ion transport in the membranes.^10–15^

For large-scale applications, such as water purification or chemical separation, inexpensive membrane manufacturing methods must be developed.^16,17^ Layer-by-layer deposition (LbL) of polyelectrolytes offers such a solution.^18^ LbL deposition is a bottom up approach that has been leveraged to create a range of functional materials including reverse osmosis membranes,^19,20^ polymer/clay fire retardant coatings,^21,22^ nanoparticle electrocatalysts^23,24^ and Metal–Organic Frameworks (MOFs).^25,26,27^ In its simplest form, a polyelectrolyte consists of an anionic polymer and a cationic polymer which are sequentially deposited on a substrate, forming one “bilayer” (BL). Aqueous solutions of inexpensive polymers are often chosen and substrates are simply LbL dip coated to form the polyelectrolyte films.^28,29^

A variety of substrates have been used for polyelectrolyte fabrication, including anodic alumina,^30,31^ mesoporous silica,^32^ inverse opal structures,^33^ quartz nanopipettes,^34^ and ion-tracked polymeric membranes,^35–40^ among others. The intrinsic surface charge found on most substrates allows for direct dip coating without extensive surface preparation, though some groups have specially prepared surfaces, often amine-terminated.^30,37,41,42^ As BLs are added to a planar surface, film thickness can grow exponentially^43^ or linearly^28,50^ depending on deposition conditions. Polyelectrolyte film growth on a nanoporous surface, however, is more complex, with formation of a dense gel in the nanopore governed by pore size, ionic strength, and the specific chemistry and molecular weight of the polymer used.^38,40,44^

Commonly, polymers containing either amines or sulfonate groups are used as cooperative elements to form the self-assembled polyelectrolyte BLs.^30,35,36,45,46^ The positive charge of the amine, complemented by the negative charge of the sulfonate, enables LbL assembly *via* electrostatic and hydrogen bonding attractions. Less commonly used are anionic polymers containing carboxylic acids.^34,43,46^ In the present work, the electrostatic interactions of amines and carboxylic acids are exploited to facilitate the LbL assembly, along with the fact that these moieties can be chemically cross-linked to modify the properties and functionalities of the LbL coatings. These carboxylic acids present opportunities for a range of cross-linking options, including carboxylic acid-amine coupling to form amide bonds, or amine-to-amine cross-linking *via* glutaraldehyde (GA). Even less explored are how these cross-linking chemistries influence both the ionic selectivity and ionic conductivity through nanoporous membranes, enabling further refinement of ionic transport properties for targeted applications.

In this work, LbL deposition is applied to create nanoporous polymer membranes 64 cm^2^ in area coated in polyelectrolytes of poly(acrylic acid) (PAA) and poly(ethyleneimine) (PEI). In fact, the polyelectrolyte film assembly is described, and ionic selectivity, ionic conductivity, and chemically stabilized film integrity are demonstrated to be further tuned by the choice of cross-linking agent and the influence it has on overall charge in the polyelectrolyte film, an aspect relatively unexplored in the literature. These results are paramount for industries that need inexpensive ion-selective membranes, such as energy storage *via* fuel cells or flow batteries, and water purification by electrodialysis.

## Experimental

### Membrane synthesis

All chemicals were purchased and used without further purification. Sodium chloride (NaCl, ACS reagent grade >99.0%, Sigma-Aldrich), poly(acrylic acid) (PAA, 35 wt% in H_2_O, average *M*_w_ = ∼100 000, Sigma-Aldrich), polyethylimine (PEI, branched, average *M*_w_ = ∼25 000, Sigma Aldrich), glutaraldehyde (GA, 25% solution in H_2_O, Sigma-Aldrich), *N*-(3-dimethylaminopropyl)-*N*′-ethylcarbodimide hydrochloride (EDC, commercial grade, Sigma-Aldrich) and sodium hydroxide (NaOH, >98%, Fisher Scientific). All water was deionized and purified to 18.2 MΩ cm.

Track-etched nanoporous polycarbonate (PC) support membranes (0.05 μm pore, 90 mm, Sterlitech Corporation) were first treated to remove a thin polyvinyl pyrrolidone (PVP) layer. The membranes were etched in a 4.5 M NaOH solution for 5 minutes, followed by rinsing in DI water. Then they were treated with UV–ozone (UVO-Cleaner model 144A, Jelight Company Inc.) for 10 minutes on each side. Immediately thereafter, the membranes were immersed in the 0.1 wt% PEI solution (pH = 10.4) for 5 minutes, rinsed in DI water, then immersed in the 0.2 wt% PAA solution (pH = 3.2) for 5 minutes. This process constituted the 1^st^ bi-layer (BL) of the LBL assembly process. Subsequent BLs were then assembled by dipping in the polymer solutions for only 1 minute each. The self-assembled polyelectrolyte BLs were then tested as made or cross-linked with either GA or EDC. The GA cross-linked membranes were immersed in a 25% GA solution for 12 hours and then washed with copious amounts of DI water. The EDC cross-linked membranes were immersed in a 100 mM EDC solution in water for 12 hours and then washed with copious amounts of water. From these 90 mm diameter membranes, daughter membranes 20 mm in diameter were punched out for all subsequent testing.

### FTIR and SEM characterization

Fourier transform infrared spectroscopy (FTIR) was done using a Thermo Nicolet NEXUS 870 FTIR ESP equipped with a PIKE Technologies MIRacle Attenuated Total Reflection (ATR) system with a diamond/ZnSe crystal.

Scanning electron microscopy (SEM) was performed using a Zeiss GeminiSEM 500 at 2–5 kV accelerating voltage and 3–5 mm working distance. A 1–3 nm layer of Au–Pd sputtered onto the samples to minimize the effects of sample charging. Cross-sections of PC membranes were obtained by a freeze–fracture method where the membranes were frozen at cryogenic temperatures in water, forming an ice block that could then be snapped in two. The membrane was warmed to room temperature and dried under nitrogen before interrogation in SEM.

### Electrochemical measurements

Ionic selectivity measurements were performed following a potentiometric method described in depth elsewhere.^9^ Briefly, each membrane was sealed in a U-shaped cell equipped with Luggin probes positioned 1 cm from the membrane, and a Ag/AgCl wire placed in each Luggin probe. The ground lead of the electrometer was always placed in the “right” side of the U-cell while the working lead was always placed in the “left” side. Both sides were filled with 0.1 mM aqueous NaCl and the potential, measured by HP 34401A multimeter, was allowed to equilibrate to 0 ± 2 mV. The concentration of aqueous NaCl on the left side of the membrane was then fixed at 0.1 mM, while the concentration on the right side was varied from 0.1 mM to 1 M (all NaCl concentrations were sequentially diluted from a 1 M stock solution). At each concentration, the voltage was allowed to stabilize. Then the cell was rinsed and equilibrated to 0.1 mM NaCl. Then the sides were switched, with a constant 0.1 mM aqueous NaCl on the right side of the cell. Before use, all solutions were allowed to equilibrate with laboratory atmosphere, stabilizing at pH = 6.0. Ionic selectivity measurements were performed in quadruplicate on different samples punched out of the same mother membrane.

Membrane conductivity was evaluated using a different test cell and a procedure described elsewhere,^47^ where membranes were soaked in 10 mM or 100 mM NaCl for at least 72 hours and then stacks of 1–4 membranes were sequentially measured in a Swagelok-style cell, fundamentally different from the one used to measure selectivity. Here membrane stacks were physically contacted with 316 steel discs with no excess bulk electrolyte in the test cell. Electrochemical Impedance Spectroscopy (EIS) was used to interrogate the impedance of the membrane stacks using a Solartron ModuLab Materials Test System where a 10 mV RMS AC was applied at 0 V *vs.* the open circuit potential over a 1 MHz to 10 Hz frequency range. The membrane stack resistance was recorded as the real impedance value taken where the imaginary impedance was zero.

## Results and discussion

**Scheme 1:**
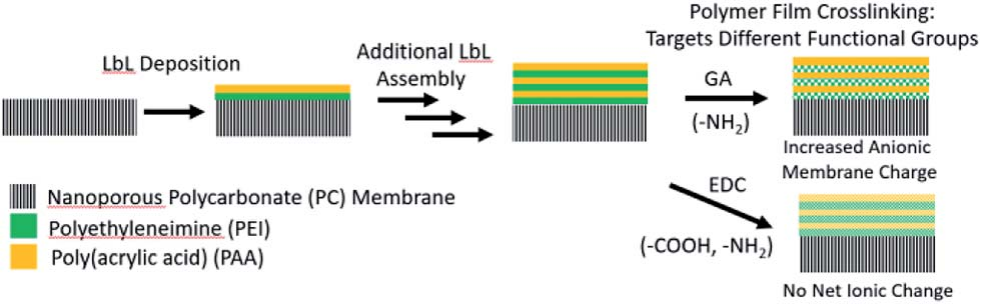
Schematic of the layer-by-layer (LbL) deposition process where polymer layers are sequentially deposited onto the nanoporous membrane and subsequently cross-linked with agents targeting different functional groups.

The LbL deposition of the polyelectrolyte thin films was accomplished by dip coating the nanoporous PC membranes in aqueous solutions containing the dissolved polymer constituents. Scheme 1 illustrates the LbL deposition process, where the nanostructured polyelectrolyte layers are built up one layer at a time. After the desired number of layers was deposited, the polymers were cross-linked using GA or EDC to tune further the charge density and to increase the stability of the polyelectrolyte. GA is known to cross-link primary amines, thus decreasing the amount of cationic fixed charge in the membrane.^48^ EDC, on the other hand, links primary amines to carboxylic acids, decreasing both the cationic and anionic fixed charge by the same amount.^49^ To best understand how the film thickness and cross-linking influence the resulting film morphology, ionic conductivity, and ionic selectivity, a series of membranes were synthesized. Membranes with either 1, 3, or 5 BLs were dip coated and cross-linked with GA, and are hereafter referred to as “1BL GA”, “3BL GA”, and “5BL GA”. Similarly, membranes were synthesized with 3BL, but not cross-linked (3BL Not X-linked), or 3BL and EDC cross-linked (3BL EDC).

**Fig. 1:**
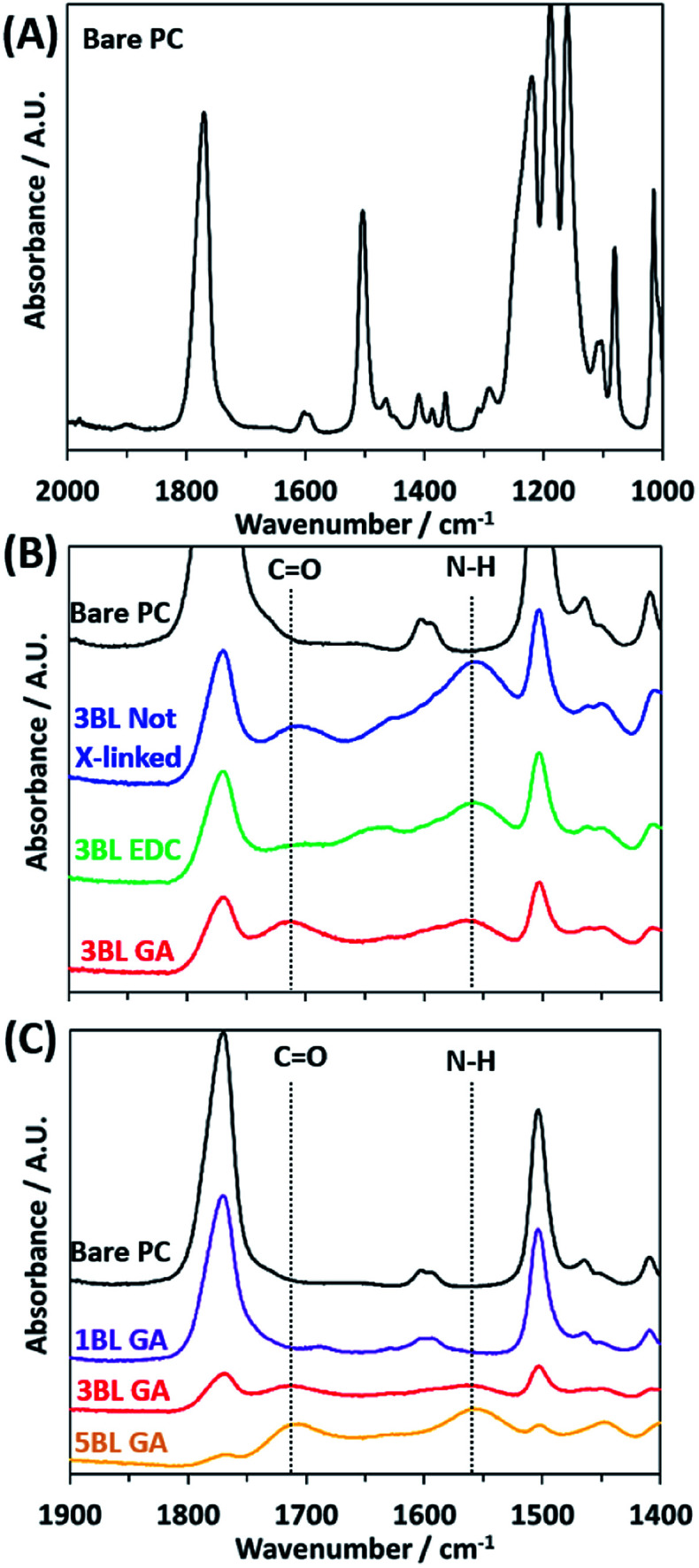
FTIR spectra of the bare polycarbonate (A) and polyelectrolyte on polycarbonate membranes showing the effect of cross-linking agent (B) and increasing number of bilayers (C). The positions of the carboxylic acid C=O stretch and the primary amine N–H bend are shown with the dotted lines in each.

FTIR absorbance was used to verify deposition of the polyelectrolyte layers and efficacy of crosslinking. FTIR spectra were recorded for all samples, as made and after crosslinking, and are plotted in Fig. 1. The bare PC spectrum reveals the peaks characteristic to the carbonate C=O stretch at 1769 cm^−1^, C–H bend at 1502 cm^−1^, and multiple bands around 1216–1157 cm^−1^ corresponding to the ether C–O stretches. Upon coating the PC support with polyelectrolyte, the peaks associated with the PC support membrane decrease in intensity while two main peaks of interest appear, labeled “C=O” and “N–H” in Fig. 1B and C. These two peaks are assigned to the carboxylic acid C=O stretch in PAA at 1714 cm^−1^ and the N–H bend from PEI at 1550 cm^−1^.

The degree of cross-linking can be observed from the FTIR spectra. In Fig. 1B the FTIR spectra for the 3BL Not X-linked sample shows the “N–H” peak has a larger absorbance than the “C=O” peak. Upon crosslinking with EDC, the “N–H” and “C=O” absorbance peaks decrease in intensity, but the decrease is similar between the two peaks, indicating that the EDC has lowered the number of amine and carboxylic acid functional groups in the polyelectrolyte by equal amounts. However, the 3BL GA cross-linked spectra shows the “N–H” peak has decreased substantially relative to the “C=O” peak, indicating a large decrease in the number of amine groups in the polyelectrolyte. This pattern indicates that the cross-linking was effective in changing the relative ratios of the functional groups, thus changing the resulting charge density present in the polyelectrolyte.

Fig. 1C shows how the two peaks associated with the layered polyelectrolyte increase in intensity with respect to the PC peaks as the number of BLs increases, with GA cross-linking. The peak associated with the “N–H” peak continues to increase with increased BL deposition. This increase indicates that the amines in the polyelectrolyte have not been removed fully during cross-linking. This deficiency is most likely because the cross-linking reaction necessitates the amines to be in close physical proximity to each other and that there are some that cannot be cross-linked effectively due to the randomized locations of the functional groups in the film upon deposition.

**Fig. 2:**
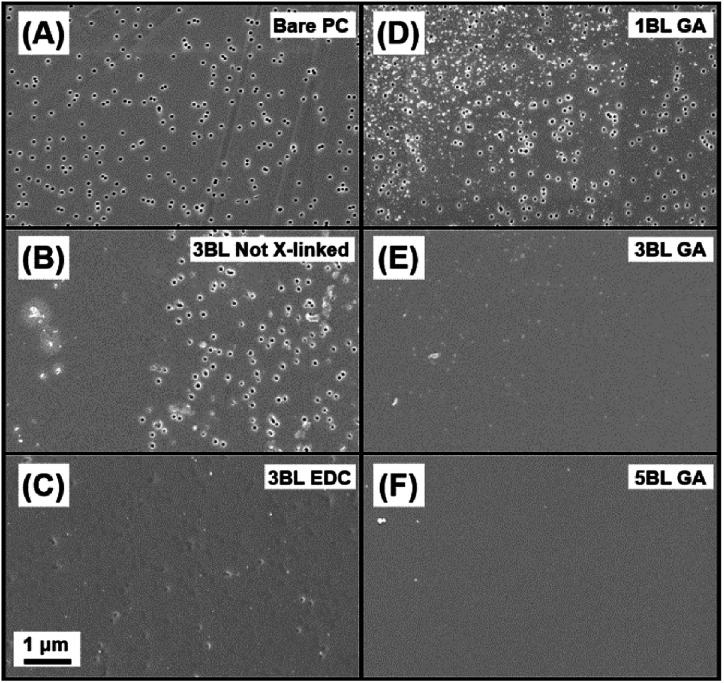
SEM plan view images of the polyelectrolyte coated membranes. (A) Bare polycarbonate, (B) 3BL not cross-linked, (C) 3BL EDC, (D) 1BL GA, (E) 3BL GA and (F) 5BL GA. All images have the same scale bar.

If any pores are not coated with polyelectrolyte, the resulting properties of the membrane will be affected. A uniform coating of polyelectrolyte is important for improving the membrane's ionic selectivity and was only achieved using both cross-linking and film thicknesses of at least 3BL. Plan view and cross-sectional SEM images were taken to determine relative uniformity and thickness of the self-assembled polymers layers. Fig. 2 shows the resulting topologies of each membrane type, with a bare PC membrane presented for comparison in Fig. 2A. Fig. 2B and C show 3BL Not X-linked and 3BL EDC, respectively. 3BL Not X-linked shows evidence that the polymer was only loosely bound to the support membrane where large portions of the membrane reveal exposed pores while other areas of the same membrane are covered. It is likely that the polymers have slowly diffused along the membrane surface or re-dissolved into solution, leading to exposed pores in large portions of the membrane. However, the images of “3BL EDC” do not show exposed nanopores and the film looks smooth and uniform, confirming that cross-linking of the polyelectrolytes is imperative for overall film adhesion and chemical stability.

Fig. 2D, E and F present plan views of 1BL GA, 3BL GA, and 5BL GA, respectively. 1BL GA still has many of the nanopores exposed, but the polymer coating is already starting to fill the pores and coat the surface. By 3BLs (GA crosslinked), the pores are completely covered and the film is largely uniform. By 5BLs (GA crosslinked), the membrane appears to be the smoothest and most uniform, even macroscopically. Thus, it is concluded that films at least 3BL thick along with chemical crosslinking for polyelectrolyte film adhesion are necessary to completely cover the nanopores.

**Fig. 3:**
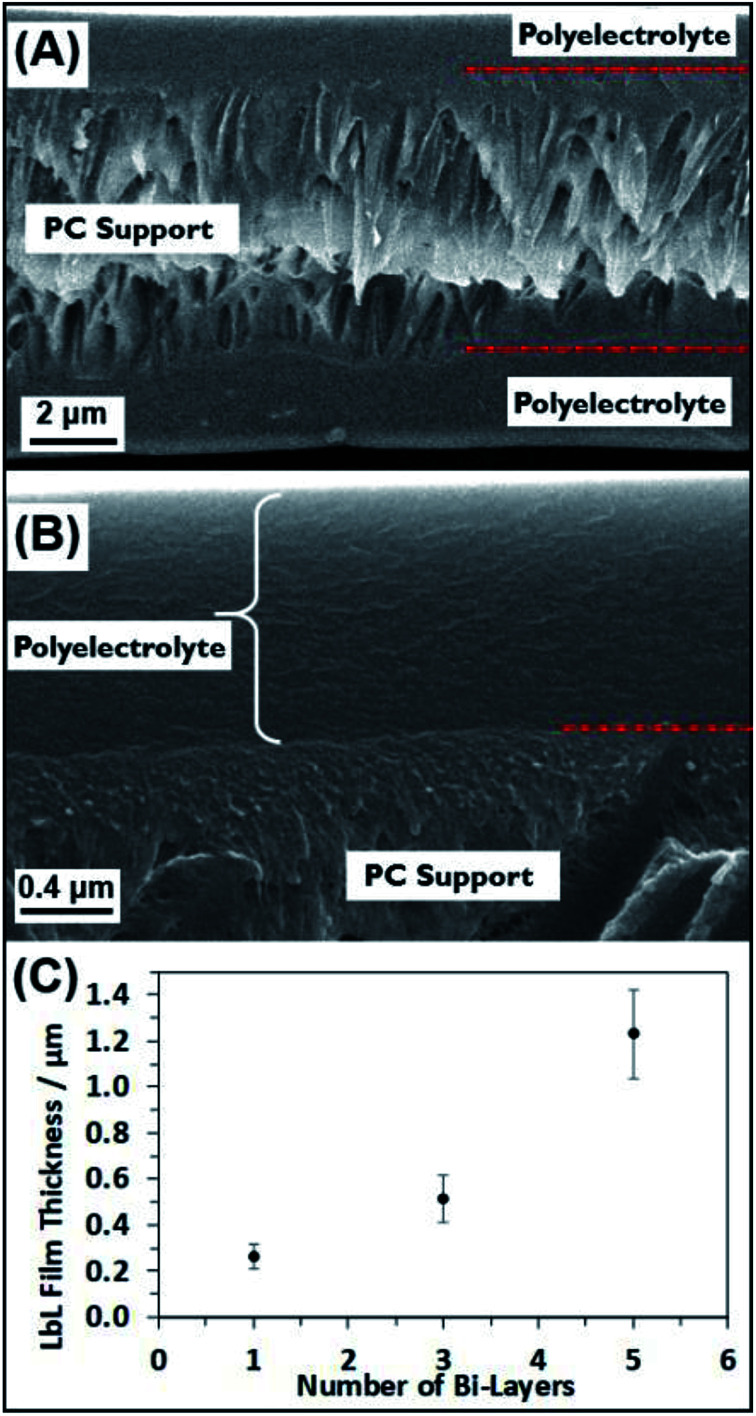
Cross-sectional SEM images of some membranes used to calculate the thickness of the coated polyelectrolyte layers. (A) 5BL GA and (B) is a magnification of the top layer of polyelectrolyte seen in (A). Red dotted lines denote the interface between the polyelectrolyte and the PC support membrane. (C) Plot of the measured thicknesses of the polyelectrolyte layers. Error bars in (C) represent one standard deviation.

To gain further understanding of the self-assembled polyelectrolyte coating structure and thickness, cross-sectional analysis was performed. Fig. 3A and B show the cross section of a 5BL GA cross-linked sample. The cross section shows the PC membrane in the middle, sandwiched between the two layers of polyelectrolyte resulting from the coating of both sides of the PC membrane. In Fig. 3B, the interface between the PC support membrane and the self-assembled polyelectrolyte suggests coherent interaction between the two polymers. A plot of the thicknesses of the deposited polyelectrolyte can be seen in Fig. 3C. As the number of bilayers increases, the thickness increases, as expected. Measuring the thickness of the polyelectrolyte on the PC membrane at different points determined the thickness of the 5BL GA membrane to be 1230 ± 193 nm, on average. Similar thickness results were seen from previous reports,^50^ where thick polymer films resulted with PEI and PAA solutions.

**Fig. 4:**
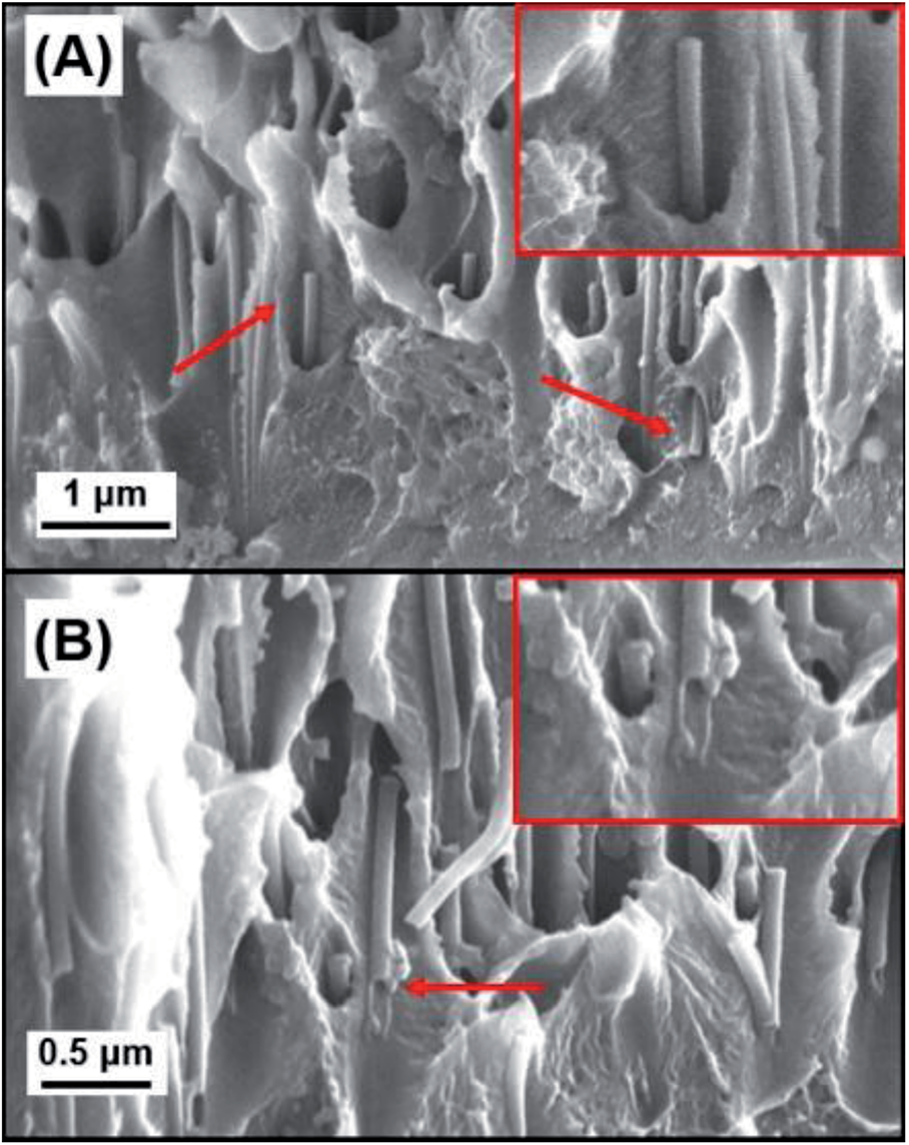
(A) and (B) Cross-sectional SEM images of 3BL GA membranes showing filled pores where the polyelectrolyte is forming “nanowire” like structures coming out of the PC membrane. Red arrows in (A) point to the emergence of the polyelectrolyte “nanowire” (magnified view shown in corresponding inset) and (B) shows the remnants of filled nanopores, where some are pulled apart and appear hollow (magnified view shown in corresponding inset).

Additional cross-sectional SEM images of a coated membrane can be seen in Fig. 4. This membrane was coated with a 3BL GA cross-linked polyelectrolyte film and there is clear evidence of the polyelectrolyte filling the PC nanopores. Fig. 4A shows many of the now exposed pore interiors with a “wire” or “rod” like structure protruding from the inside of the membrane. These structures were not visible in the bare PC and the 1BL GA (ESI Fig. S1†) did not have any obvious filling in the pores, although 1BL GA had what looked like some tube formation inside the pores but was not obvious. Some evidence for tubular structures was observed in the SEM images. Fig. 4B shows a pore filling structure that was ripped open by the freeze–fracture process and appears largely hollow on the inside. The concentric filling of the pores forming hollow tubes that then completely fill upon more additional BL dip coatings is likely, but unconfirmed. These tubes were also not observed in the uncoated bare PC membrane cross sectional images (ESI Fig. S1†). The lack of tubes of wire-like structures in the uncoated membrane lead us to believe their formation is a direct result of the coating and thus are most likely polyelectrolyte.

**Fig. 5:**
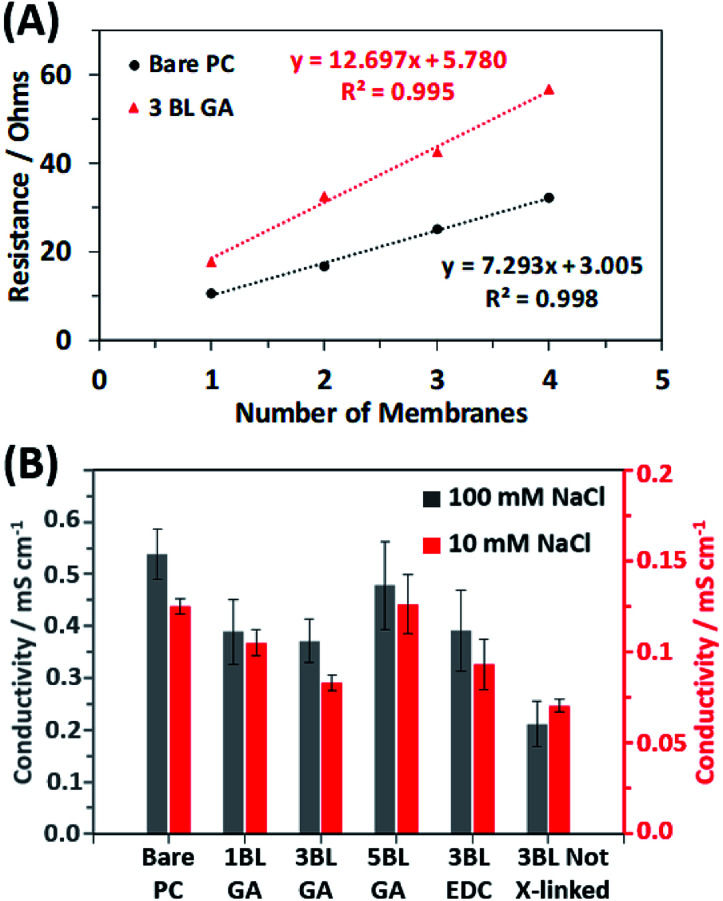
(A) Example plots of increasing total resistance with the number of membranes in a stack, with data for both samples collected in 10 mM NaCl solution. (B) The calculated conductivity of the membranes in both 10 mM NaCl and 100 mM NaCl solutions, determined from the slopes of the resistance *vs.* number of membrane plots seen in (A). The conductivities from the 100 mM NaCl solution pertain to the left axis (in black) and the conductivities from the 10 mM NaCl solution pertain to the right axis (in red).

To investigate how the resulting conductivity of the membranes is affected by the coating, the resistance of stacks of membranes cut from the same mother membrane were measured *via* impedance spectroscopy as detailed in the experimental section. Fig. 5A shows two example plots of the total resistance *vs.* number of membranes stacked together for the bare PC and the 3BL GA cross-linked membranes in 10 mM NaCl solution. The membranes were stacked and measured to obtain an average resistance per membrane, thereby eliminating contributions to resistance from the cables and fixturing. From the plots, the average resistivity of a membrane (then converted into conductivity, as discussed in the ESI†) can be calculated from the slope of the best fit line. Fig. 5B shows the calculated conductivities of all the different membrane types in both 10 mM NaCl and 100 mM NaCl. The relative changes in the conductivities between different types of membranes are the same in both NaCl concentration solutions. The membranes showed ionic conductivities that were similar magnitudes to previously examined polymer systems.^51,52^ The bare PC membranes had the highest conductivity, as expected due to the fact that no polyelectrolyte obscures the transmembrane pores. Once the 1^st^ bilayer is applied (1BL GA), the conductivity drops because the polyelectrolyte is now partially obscuring the pores, but no additional significant decrease is observed with additional coating. Once the coating is completely covering the pores, additional BLs which increase the thickness of the polyelectrolyte film, no additional decrease was observed and may have recovered some of the conductivity. Appear to increase the conductivity. This is apparent for the 5BL GA membranes where the calculated conductivity is similar to the uncoated PC membrane. Additionally, the choice of cross-linking agent used does not affect the resulting conductivity; EDC cross-linked membranes have conductivities similar to GA cross-linked membranes. However, not cross-linking the membranes, as in 3BL Not X-linked, results in the lowest conductivities out of all membranes.

**Fig. 6:**
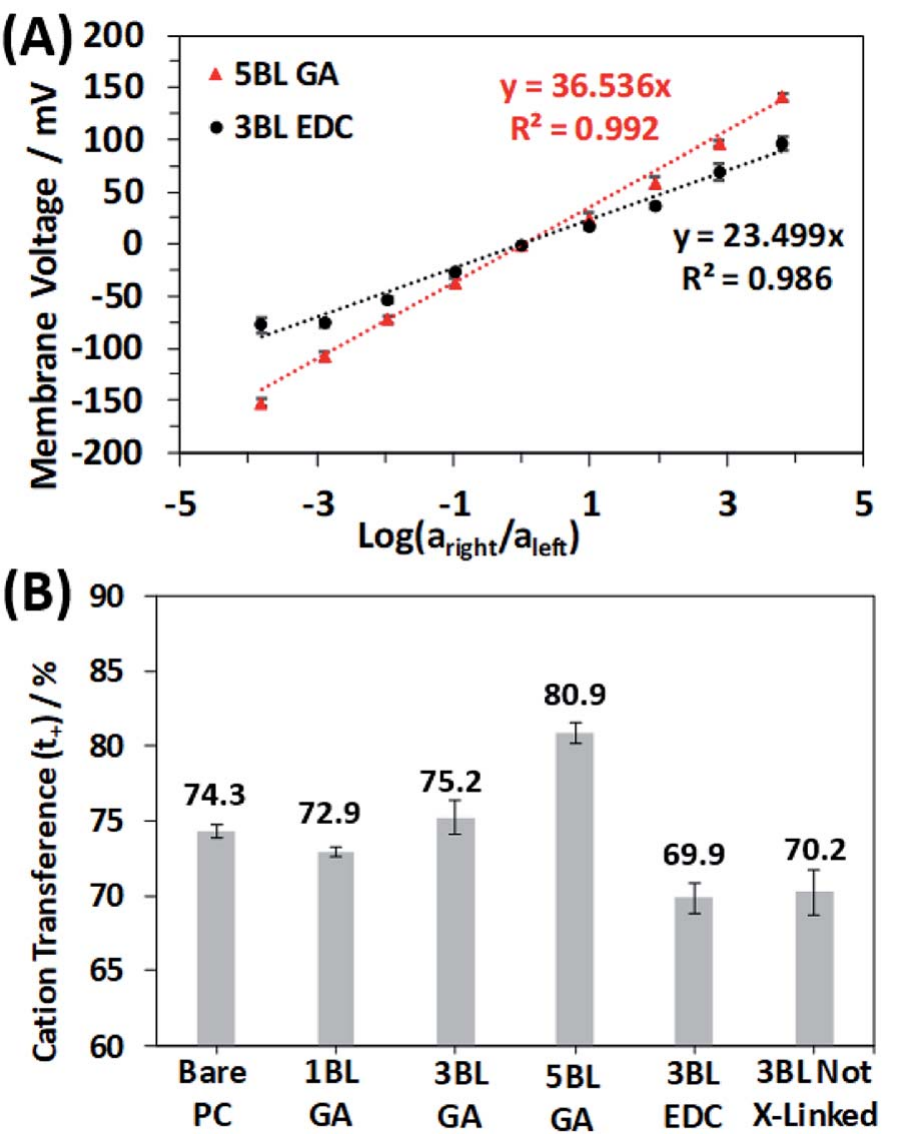
(A) Measured membrane voltage curves resulting from the selective flow of ions through the membrane. Example curves shown are the 5BL GA cross-linked membrane and the 3BL EDC cross-linked membrane to highlight the difference between a more cation selective membrane and a less cationic selective one. (B) Resulting cation transference numbers calculated from the slopes of the best fit lines in (A) for the different membranes. Error bars represent one standard deviation for both plots.

The influence of the polyelectrolyte layers on the ionic selectivity was determined from the resulting transmembrane voltage. This transmembrane voltage arises when two different concentrations of NaCl solutions are placed on opposite sides of the membrane and the diffusion of one type of ion is limited with respect to the other. The plots are linear with a positive slope, indicating a preferred cationic selective transport. The slope of the lines can be used to calculate the ion transference capability, or transference numbers. The membrane voltage, *V*_m_, for a 1 : 1 monovalent salt can be described by a modified version of the Nernst equation shown in eqn (1),^1,9^1
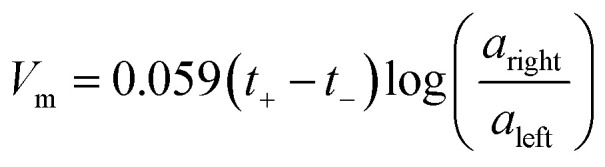
where *a*_right_ and *a*_left_ are the activities of the NaCl salt solutions placed in the right and left side of the U-cell used to measure the *trans*-membrane voltage. The NaCl concentrations were converted to activities using the well-documented activity coefficients.^53^ The cation and anion transference numbers, *t*_+_ and *t*_−_, can have values between 0 and 1 and relate the membrane's ability to transport either cations or anions selectively. A perfectly cation selective membrane would have a *t*_+_ = 1 and *t*_−_ = 0, while for a perfectly anion selective membrane *t*_+_ = 0 and *t*_−_ = 1. Therefore, *t*_+_ = 1 and *t*_−_ = 0 would yield a slope of 0.059 V, while *t*_+_ = *t*_−_ = 0.5 (*t*_+_ + *t*_−_ = 1, by definition) would yield a slope of 0, meaning the membrane has no ionic selectivity at all, since both cations and anions could diffuse through the membrane with equal currents. Negative slope would indicate anion selectivity. Fig. 6A shows example plots of the membrane voltage as a log function of the ratio of solution activities. The plots shown highlight the different slopes observed for the polyelectrolyte membranes, where the 5BL GA cross-linked membranes show the largest slope of 36.54 mV (indicating good cationic selectivity) and the 3BL EDC cross-linked membranes show a much lower slope of 23.50 mV. At high ionic strength the membrane voltage response was observed to begin to slightly deviate from the linear regime for the bare PC (ESI Fig. S4†) but deviated less for the coated membranes as seen in Fig. 6A.

All the membranes tested in this study were selective for cationic transport, but the number of deposited BLs and cross-linking type clearly influence the magnitude of the selectivity. Fig. 6B shows the results of the ionic selectivity measurements, where the cation transference number, *t*_+_, was calculated from the slopes of the transmembrane voltage curves and compiled in Table S1.† As expected, the bare PC membrane was selective for cation transport due to the anionic (COO^−^) surface charges on the entire membrane surface and pore walls. Surprisingly, the 1BL GA was less selective than the bare PC, but this quickly changed as the polyelectrolyte layers became thicker. Cation transference numbers increased by 11% as the number of GA cross-linked BLs increased from 1 to 5.

When comparing the membrane cross-linking types, there is an obvious increase in selective cation transport for the GA cross-linked membranes. This trend is expected since the GA cross-linking chemistry selectively reacts with primary amines of the PEI polymer layers and does not react with the carboxylic acid groups of the PAA. This asymmetry shifts the net fixed charge of the polyelectrolyte more negatively, increasing the preference for cations diffusing through the polyelectrolyte. Further evidence of this result is seen by comparing the selectivity of the 3BL EDC cross-linked membranes and the as made 3BL not cross-linked membranes. The selectivity of these membranes is nearly identical, which is rationalized by the fact the EDC cross-links a primary amine and carboxylic acid, causing no net change in the charge of the polyelectrolyte.

## Conclusions

The demonstrated control of ionic selectivity independent of ionic conductivity was achieved with the use of LbL deposition of many nanostructured polyelectrolyte layers and applied cross-linking agents that changed the polyelectrolyte charge density. The increased number of polyelectrolyte layers increase the selective cation transport when the polymer layers were cross-linked with GA. The conductivity decreased with the coatings, but was found to regain a portion of it upon cross-linking the polyelectrolyte. Cross-linking the membranes also increased the intermolecular integrity of the polyelectrolyte films and inhibited the slow surface diffusion and re-dissolution of the polyelectrolyte films. This study has shown how this controllable and inexpensive method can be tailored to create ion-selective and chemically robust membranes on porous supports for a wide range of applications.

## Funding sources

This work was supported by the Laboratory Directed Research and Development (LDRD) program at Sandia National Laboratories. Sandia National Laboratories is a multi-mission laboratory managed and operated by National Technology and Engineering Solutions of Sandia, LLC., a wholly owned subsidiary of Honeywell International, Inc., for the U.S. Department of Energy's National Nuclear Security Administration under contract DE-NA0003525. This paper describes objective technical results and analysis. Any subjective views or opinions that might be expressed in the paper do not necessarily represent the views of the U.S. Department of Energy or the United States Government.

## Conflicts of interest

There are no conflicts of interest to declare.

## Supplementary Material

RA-008-C8RA05580G-s001

## References

[cit1] Martin C. R., Nishizawa M., Jirage K., Kang M., Lee S. B. (2001). Adv. Mater..

[cit2] Guo W., Tian Y., Jiang L. (2013). Acc. Chem. Res..

[cit3] Daiguji H. (2010). Chem. Soc. Rev..

[cit4] Elbert J., Krohm F., Ruttiger C., Kienle S., Didzoleit H., Balzer B. N., Hugel T., Stuhn B., Gallei M., Brunsen A. (2014). Adv. Funct. Mater..

[cit5] Zhang Q., Hu Z., Liu Z., Zhai J., Jiang L. (2014). Adv. Funct. Mater..

[cit6] Liu T., Bao C., Wang H., Lin Y., Jia H., Zhu L. (2013). Chem. Commun..

[cit7] Vlassiouk I., Park C.-D., Vail S. A., Gust D., Smirnov S. (2006). Nano Lett..

[cit8] Buyukserin F., Kohli P., Wirtz M. O., Martin C. R. (2007). Small.

[cit9] Small L. J., Wheeler D. R., Spoerke E. D. (2015). Nanoscale.

[cit10] Apel P., Korchev Y., Siwy Z., Spohr R., Yosida M. (2001). Nucl. Instrum. Methods Phys. Res., Sect. B.

[cit11] Li N., Yu S., Harrell C. C., Martin C. R. (2004). Anal. Chem..

[cit12] Small L. J., Wheeler D. R., Spoerke E. D. (2014). RSC Adv..

[cit13] Cervera J., Schiedt B., Neumann R., Mafe S., Ramirez P. (2006). J. Chem. Phys..

[cit14] Siwy Z., Heins E., Harrell C. C., Kohli P., Martin C. R. (2004). J. Am. Chem. Soc..

[cit15] Kubeil C., Bund A. (2011). J. Phys. Chem. C.

[cit16] Shannon M. A., Bohn P. W., Elimelech M., Georgiadis J. G., Mariñas B. J., Mayes A. M. (2008). Nature.

[cit17] Noack J., Roznyatovskaya N., Herr T., Fischer P. (2015). Angew. Chem., Int. Ed..

[cit18] Lutkenhaus J. L., Hammond P. T. (2007). Soft Matter.

[cit19] Cho K. L., Hill A. J., Caruso F., Kentish S. E. (2015). Adv. Mater..

[cit20] Sanyal O., Sommerfeld A. N., Lee I. (2015). Sep. Purif. Technol..

[cit21] Holder K., Smith R., Grunlan J. C. (2017). J. Mater. Sci..

[cit22] Guin T., Krecker M., Milhorn A., Hagen D. A., Stevens B., Grunlan J. C. (2015). Adv. Mater. Interfaces.

[cit23] Han C., Percival S. J., Zhang B. (2016). Langmuir.

[cit24] Yaqub M., Imar S., Laffir F., Armstrong G., McCormac T. (2015). ACS Appl. Mater. Interfaces.

[cit25] Lee D. Y., Kim E.-K., Shin C. Y., Shinde D. V., Lee W., Shrestha N. K., Lee J. K., Han S.-H. (2014). RSC Adv..

[cit26] Spoerke E. D., Small L. J., Foster M. E., Wheeler J., Ullman A. M., Stavila V., Rodriguez M., Allendorf M. D. (2017). J. Phys. Chem. C.

[cit27] Robinson A. L., Stavila V., Zeitler T. R., White M. I., Thornberg S. M., Greathouse J. A., Allendorf M. D. (2012). Anal. Chem..

[cit28] Hagen D. A., Foster B., Stevens B., Grunlan J. C. (2014). ACS Macro Lett..

[cit29] Cho C., Xiang F., Wallace K. L., Grunlan J. C. (2015). Macromolecules.

[cit30] Raoufi M., Tranchida D., Schönherr H. (2012). Langmuir.

[cit31] Lazzara T. D., Lau K. A., Abou-Kandil A. I., Caminade A.-M., Majoral J.-P., Knoll W. (2010). ACS Nano.

[cit32] Brunsen A., Calvo A., Williams F. J., Soler-Illia G. J. A. A., Azzaroni O. (2011). Langmuir.

[cit33] Yeo S. J., Kang H., Kim Y. H., Han S., Yoo P. J. (2012). ACS Appl. Mater. Interfaces.

[cit34] Actis P., Vilozny B., Seger R. A., Li X., Jejelowo O., Rinaudo M., Pourmand N. (2011). Langmuir.

[cit35] Ali M., Yameen B., Cervera J., Ramirez P., Neumann R., Ensinger W., Knoll W., Azzaroni O. (2010). J. Am. Chem. Soc..

[cit36] Armstrong J. A., Bernal E. E. L., Yaroshchuk A., Bruening M. L. (2013). Langmuir.

[cit37] Chen H., Palmese G. R., Elabd Y. A. (2006). Chem. Mater..

[cit38] Alem H., Bondeau F., Glinel K., Demoustier-Champagne S., Jonas A. M. (2007). Macromolecules.

[cit39] Zao Y., Janot J.-M., Balanzat E., Balme S. (2017). Langmuir.

[cit40] Roy C. J., Dupont-Gillain C., Demoustier-Champagne S., Jonas A. M., Landoulsi J. (2010). Langmuir.

[cit41] Ali M., Schiedt B., Healy K., Neumann R., Ensinger W. (2008). Nanotechnology.

[cit42] VanDelinder V., Wheeler D. R., Small L. J., Brumbach M. T., Spoerke E. D., Henderson I., Bachand G. D. (2015). ACS Appl. Mater. Interfaces.

[cit43] Yuan W., Li C. M. (2010). Chem. Commun..

[cit44] Carrillo J.-M. Y., Dobrynin A. V. (2012). Langmuir.

[cit45] DeRocher J. P., Mao P., Han J., Rubner M. F., Cohen R. E. (2010). Macromolecules.

[cit46] Kiryukhin M. V., Man S. M., Sadovoy A. V., Low H. Y., Sukhorukov G. B. (2011). Langmuir.

[cit47] Hudak N. S., Small L. J., Pratt H. D., Anderson T. M. (2015). J. Electrochem. Soc..

[cit48] Cheung D. T., Nimni M. E. (1982). Connect. Tissue Res..

[cit49] Bax D. V., Davidenko N., Gullberg D., Hamaia S. W., Farndale R. W., Best S. M., Cameron R. E. (2017). Acta Biomater..

[cit50] Yang Y.-H., Haile M., Park Y. T., Malek F. A., Grunlan J. C. (2011). Macromolecules.

[cit51] Delongchamp D. M., Hammond P. T. (2003). Chem. Mater..

[cit52] Delongchamp D. M., Hammond P. T. (2004). Langmuir.

[cit53] CRC Handbook of Chemistry and Physics, ed. D. R. Lide, CRC Press, Boca Raton, FL, 86th edn, 2005

