# Supplementary material for: Polyelectrolyte layer-by-layer deposition on nanoporous supports for ion selective membranes
Source: RSC Adv. 2018 Sep 25;8(57):32992–9. doi: 10.1039/c8ra05580g (PMC9086297; doi:10.1039/c8ra05580g)
Supplement: RA-008-C8RA05580G-s001 [file RA-008-C8RA05580G-s001.pdf]

Electronic Supplementary Material (ESI) for RSC Advances.
This journal is © The Royal Society of Chemistry 2018

Supporting Information:

Polyelectrolyte Layer-by-Layer Deposition on Nanoporous Supports for Ion Selective Membranes

Stephen J. Percival*, Leo J. Small*, Erik D. Spoerke and Susan B. Rempe

Sandia National Laboratories, PO Box 5800, MS 1411, Albuquerque, NM, USA 87185

Additional SEM images of the membrane cross sections presented in Figure S1 show cross sections of the membranes with no coating (bare PC), 1BL GA crosslinked, 3BL GA crosslinked and 5BL GA crosslinked in Figures S1A, S1B, S1C and S1D respectively. The uncoated bare PC membrane has no evidence of pore filling and the 1 BL coated membranes shows very little evidence of polyelectrolyte filling the pores. The polyelectrolyte nanowires can be seen emerging from the polycarbonate membrane for the 3 and 5 BL coated membranes, some of which have red arrows pointing at them for ease of identification. Also observed in the 3 BL membranes was what appeared to be polyelectrolyte precipitate inside of the pores. This precipitate may be part of the pore filling process where as the membrane has more BLs applied to it the precipitates will grow and coalesce into a filled pore structure.

Figure S2 shows additional SEM images of a membrane that was coated with 3 BLs but not crosslinked. Large whitish patches can be seen in the image shown in Figure S2A surrounded by a uniform surface. The area denoted by the red box is magnified in Figure S2B where it is revealed that the white patches are uncovered nanopores. These uncovered pore "patches" are observed only on the as made not crosslinked membranes and were not observed on the crosslinked membranes.

The intercept of the Nyquist plots with the real impedance axis (i.e where the imaginary portion was zero) was the value used in determining the resistance for a given measurement of the test cell and a given number of membranes. From the resistances of the different numbers of membranes the values were plotted versus membrane number and the slope used to calculate the membrane conductivity (slope = resistance/membrane) and the y-intercept taken as the intrinsic resistance of the test cell and contacts. Figure S3 shows some example Nyquist plots for bare PC membranes with different number of membranes, from 1 to 4 membranes. Only the relevant high frequency portion of the impedance spectra are shown. As the number of membranes stacked together increases the impedance spectra shifts to higher values where the intercept is

crossed. The values obtained from the impedance intercepts could then be plotted and the slope of the line used to calculate the conductivity using the equation shown below.

$$Conductivity\ (mS * cm^{-1}) = \frac{total\ membrane\ thickness\ (cm)}{\{(Slope\ of\ the\ line\ (Ohms/membrane)) * (Area\ of\ the\ membrane\ (cm^2))\}} * 1000$$

The membrane thickness was measured from the SEM cross-sections and was the total thickness of the PC support membrane plus the added polyelectrolyte (which coated both sides). The thickness obviously became thicker with increase BL number and the area of the membranes was the same for all membranes, determined by the size of the punch used to punch out test membranes (0.7125 cm^2).

Figure S4 shows the selectivity test as described in the experimental section for the bare polycarbonate membranes where the membrane voltage changes with the different ionic strength solutions. The line deviates from the linear regime observed at low ionic strengths and shows a lower potential increase with increasing ionic strength. This observation shows that the pores have a higher selectivity at lower ionic strength solutions that at higher. This phenomenon was also observed for the polyelectrolyte coated membranes but the deviation from the linear region is much less pronounced leading.

Table S1 lists all the values for the slopes of the trans-membrane voltages curves for the different membranes, the corresponding (t_+ - t_-) values, and the cation transference number (t_+) values calculated from the ion selectivity measurements. The values for the cation transference number are plotted in the bar graph seen in Figure 7 of the main text.

Figure S1: SEM image of the membrane cross sections showing the pore filling of the polyelectrolytes: (A) a bare PC membrane with nothing observed in the pores, (B) a 1BL GA crosslinked membrane with very little evidence of pore filling, (C) a 3BL GA crosslinked membrane with pore filling and (D) a 5BL GA crosslinked membrane also with pore filling. Red arrows in (D) point to the polyelectrolyte "wires" and the inset shows a magnified view of a few of the "wires".

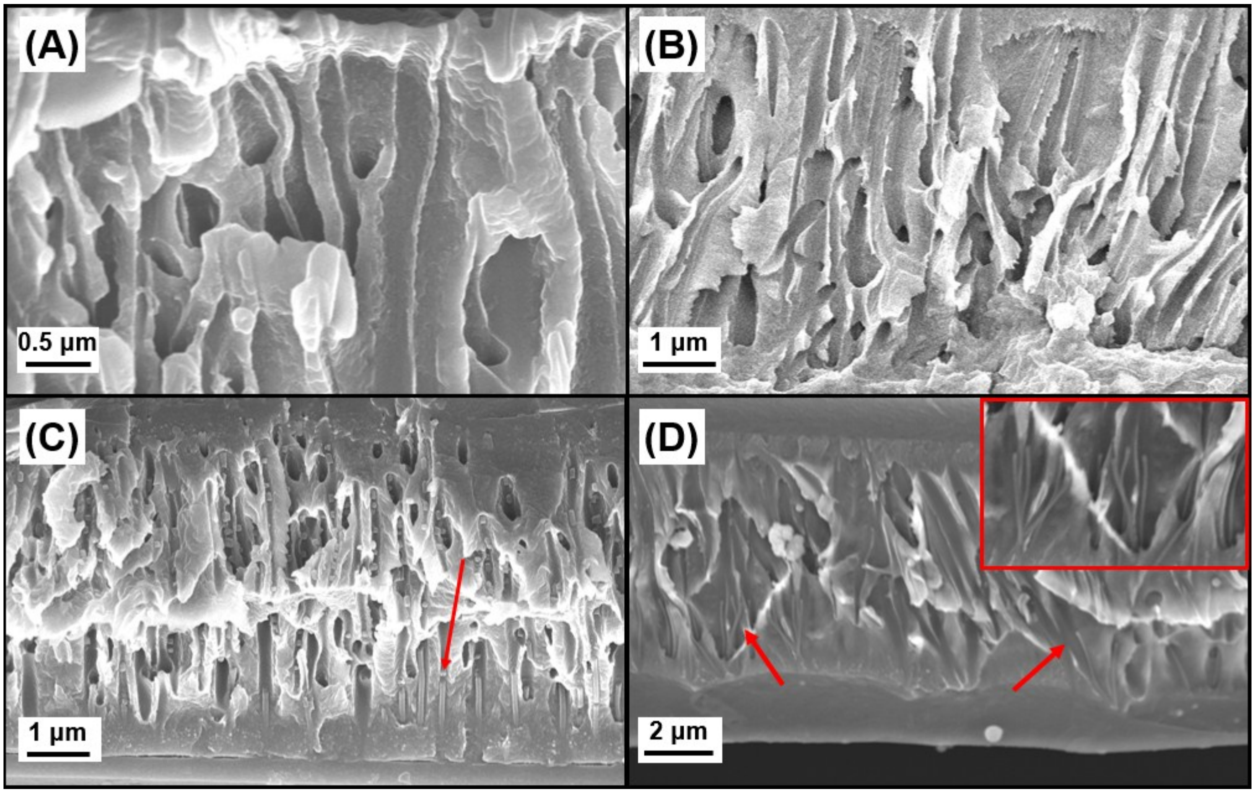

Figure S2: (A) SEM of a as made not crosslinked membrane with 3 BLs of polyelectrolyte on it showing large areas of the membranes that are not coated with polyelectrolyte. (B) shows the magnified view of the region in (A) outlined with thea red box.

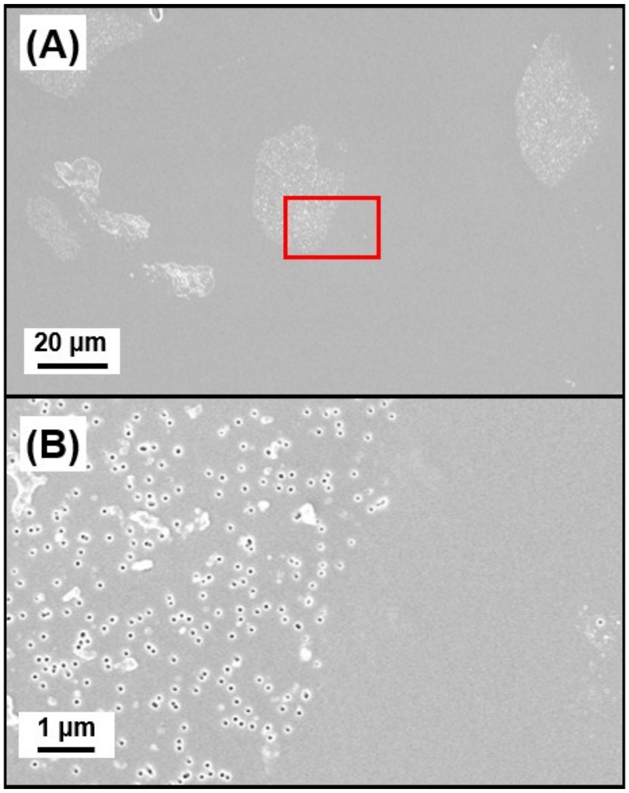

Figure S3: Impedance measurement Nyquist plots from different numbers of bare PC membranes used to determine the membranes conductivities. The arrows point to the impedance spectrum for the given number of stacked membranes, from 1 to 4 membranes. The blue dot and red dot for each spectrum denotes the 1MHz and 20 kHz frequency measurement points respectively. All impedance spectra taken after soaking membranes in 10 mM NaCl solution.

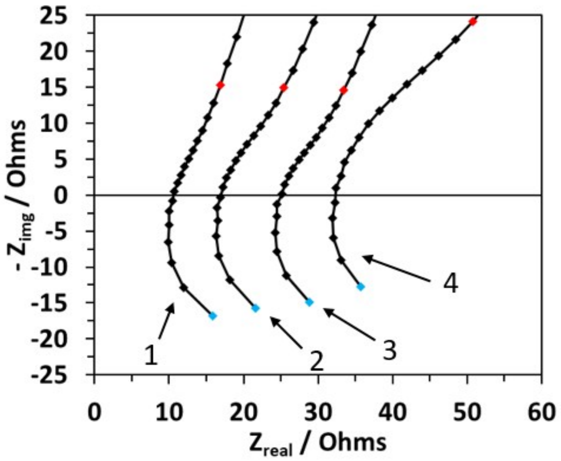

Figure S4: Membrane voltage response of bare PC membranes at different ionic strengths. At the highest ionic strength solutions, the membrane voltage is observed to begin to deviate from the linear best fit line and shows a smaller change than in low ionic strength solutions.

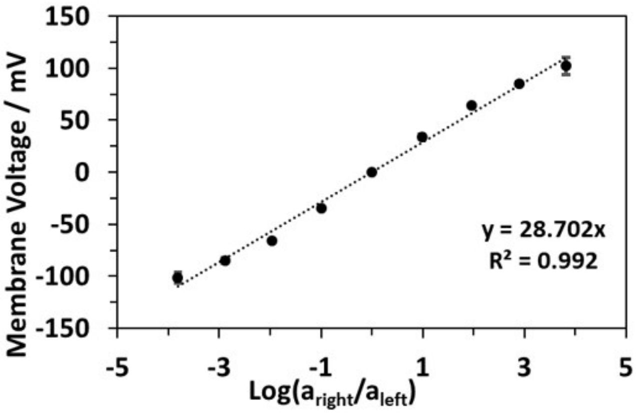

Table S1: Calculated polyelectrolyte membrane cation transference numbers taken from the slope of the membrane potential curves seen in Figure 6. Number of samples tested for each membrane type is 4.

	Slope of Line	$(t_+ - t_-)$ / %	t_+ / %
Bare PC	28.7 ± 0.527	48.5 ± 0.89	74.3 ± 0.44
1BL GA	27.1 ± 0.341	45.9 ± 0.57	72.9 ± 0.28
3BL GA	29.8 ± 1.30	50.4 ± 2.22	75.2 ± 1.10
5BL GA	36.5 ± 0.827	61.8 ± 1.40	80.9 ± 0.70
3BL EDC	23.5 ± 1.19	39.7 ± 2.01	69.9 ± 1.01
3BL NOT Crosslinked	23.9 ± 1.74	48.7 ± 2.94	70.2 ± 1.47
